# Self-reported continuity and coordination of antenatal care and its association with obstetric near miss in Uasin Gishu county, Kenya

**DOI:** 10.4102/phcfm.v15i1.3452

**Published:** 2023-01-25

**Authors:** Samuel M. Mulongo, Doreen Kaura, Bob Mash

**Affiliations:** 1 Department of Nursing, Faculty of Medicine and Health Sciences, Stellenbosch University, Cape Town, South Africa; 2 Department of Family and Emergency Medicine, Faculty of Medicine and Health Sciences, Stellenbosch University, Cape Town, South Africa

**Keywords:** longitudinal continuity, relational continuity, parallel coordination, sequential coordination, obstetric near miss

## Abstract

**Background:**

Continuity and coordination of care are core principles of high-quality primary health care. Optimising continuity and coordination improves maternal satisfaction. However, their association with morbidity and mortality outcomes is unclear. The obstetric near-miss approach can be used to investigate whether continuity and coordination influences the occurrence of a severe maternal outcome.

**Aim:**

To compare self-reported continuity and coordination of care between obstetric near-miss survivors and those without near miss during pregnancy, delivery and postpartum.

**Setting:**

Uasin Gishu county, Rift Valley region, Kenya.

**Methods:**

A cross-sectional survey targeting 340 postnatal mothers. Continuity of care index (COCI) and modified continuity of care index (MCCI) were used to estimate longitudinal continuity. The Likert scale was administered to measure perceived continuity and coordination of care. Mann–Whitney *U* test and binomial logistic regression were used for hypothesis testing.

**Results:**

COCI and MCCI were lower among near-miss survivors (COCI = 0.80, *p* = 0.0026), (MCCI = 0.62, *p* = 0.034). Near-miss survivors scored lower on items assessing coordination between a higher-level provider and usual antenatal clinic (mean = 3.6, *p* = 0.006) and general coordination of care during pregnancy (mean = 3.9, *p* = 0.019). Presence of a non-life-threatening morbidity in pregnancy was associated with occurrence of near miss (aOR = 4.34, *p* = 0.001).

**Conclusion:**

Near-miss survivors scored lower on longitudinal continuity and coordination of care across levels. Further research should focus on strengthening coordination, determining the optimal level of longitudinal continuity and improving systems for early identification and management of morbidities in pregnancy.

**Contribution:**

The results of this study show that while longitudinal and relational COC is important during the antenatal period, the presence of a non-life-threatening condition in pregnancy remains the most important predictor of the occurrence of a near miss.

## Background

In low- and middle-income countries (LMICs), a significant proportion of maternal deaths because of pregnancy-related factors may be attributed to failures in continuity and coordination of care.^1^ Continuity and coordination are among the five core principles of high-quality primary health care yet are under-researched in LMICs.^2,3^ Improvement in continuity and coordination has the potential to reduce fragmentation of care, improve utilisation of health services, save costs and improve health systems performance.^4^

According to World Health Organization (WHO) (2016), *longitudinal continuity* is the degree to which a client attends the same provider across time.^2^ In the context of antenatal care (ANC), this may represent the same individual health care provider, team of providers or antenatal clinic. *Interpersonal (relational) continuity* entails the quality of relationship between a client and their provider. Such a relationship should be characterised by increasing trust, mutual respect and knowledge of the person. *Informational continuity* requires the availability of a client’s medical information at every visit and across time. Coordination entails the collaboration of professionals to provide seamless care across boundaries. Two distinctions are made, *sequential coordination* (collaboration across facilities or levels of care) and *parallel coordination* (collaboration within facilities or the same level of care).^2^ It should be noted that these concepts are interrelated and not isolated.

A recent focus in maternal health services is how to optimise care continuity models for positive pregnancy outcomes.^5,6^ Continuity is assumed to foster better relationships and more in-depth knowledge of the expectant woman, which allows the provider to move faster in the consultation (more efficient), know what is important to focus on (better quality of care) and be more informed of all the issues if the person is referred (coordination). There is evidence that longitudinal and interpersonal continuity improves expectant women’s satisfaction and other intermediate outcomes such as early presentation to antenatal clinics^7,8^ and prevention of stigma and suicidal ideation.^9,10^

Nevertheless, evidence for the effect of continuity on maternal morbidity and mortality is mixed. Furthermore, there appear to be regional differences in the level of continuity necessary for optimal delivery outcomes. In Ghana, high longitudinal continuity indices of 0.80–0.90 during the antenatal period were associated with fewer caesarean and instrumental deliveries.^11^ On the other hand, in Netherlands and Belgium, moderate longitudinal continuity indices of 0.40–0.50 were associated with lower levels of caesarean sections and complicated deliveries.^12,13,14^

This difference may be attributed to variations in maternal health delivery systems. In the Netherlands, for example, midwives usually transfer surveillance of expectant women to physicians and obstetricians during the third trimesters, thus explaining the low continuity indices. In Ghana and many low-income settings, midwives may be responsible for surveillance up to the time of delivery, explaining the high continuity indices. The former requires a higher level of coordination characterised by communication and collaboration between providers of different cadres and at different levels. The latter, which favours a more personalised continuity approach, may support long-lasting therapeutic relationships and ease of communication because of familiarity between provider and client. It appears therefore that achieving the right level of continuity necessary for building a meaningful provider–mother relationship while ensuring coordination within and between levels is of interest regardless of context. This places care coordination as a central theme when studying the effect of longitudinal and relational continuity on maternal outcomes.

Strengthening coordination of care alongside continuity may improve maternal health outcomes such as increased birth weights^15^ and reduced hospitalisations.^16^ A Cochrane review identified eight coordination interventions in maternal health that have been implemented in low-income countries with promise. These include the use of multidisciplinary clinical pathways, strengthening interactive communication between nonspecialists and specialists, integration of services or service elements and use of midwifery teams, among others.^17^

Kenya, like many LMICs, emphasises completion of at least four and preferably eight antenatal visits under the focused antenatal care (FANC) model. Ideally, a woman is expected to attend only one facility, unless a complication necessitates referral. However, recent evidence suggests that expectant mothers in Kenya may be switching facilities more, with the average woman now attending 2.6 facilities across pregnancy.^18^ Furthermore, because of plurality in the health sector, expectant women now have more choices of facilities, including private and church-based facilities. It is, however, unknown whether the resulting fragmentation has a negative impact on morbidity and mortality outcomes.

Studies that assess the association between continuity and coordination, and morbidity or mortality outcomes are concentrated in high-income countries,^5,15^ predominantly use insurance claims data^11,13,16^ and focus on intermediate outcomes.^15,18,19^ A potentially useful way to relate continuity and coordination with morbidity and mortality outcomes is to utilise the near-miss approach. This approach compares women who almost die but survive a life-threatening condition during pregnancy, labour or delivery against those without life-threatening conditions. Increasingly, the near-miss approach is used for assessing the quality of maternal health care because the study population is larger than for maternal deaths, and there is the possibility of interviewing survivors.^20,21^

This study aimed to investigate whether self-reported continuity and coordination of care are associated with the occurrence of a near miss. We hypothesised that women who experienced a near miss during pregnancy or childbirth will score lower on measures of continuity and coordination. We focused on four important aspects, namely: (1) longitudinal continuity, (2) relational continuity, (3) informational continuity and (4) sequential coordination.

## Methods

### Study design

This case–control study was part of a larger explanatory sequential mixed methods study^22^ aimed at evaluating continuity and coordination of care among obstetric near-miss cases at a tertiary hospital in the Rift Valley region of Kenya. The larger study was carried out in four phases: phase one examined determinants of obstetric near miss in the hospital under consideration. The second phase (the current study) compared continuity and coordination in obstetric near misses with normal deliveries using a cross-sectional survey. The third phase qualitatively assessed continuity and coordination among near-miss cases. The fourth phase involved the integration of findings from the quantitative and qualitative phases.

### Setting

Kenya has six levels of care, ranging from household-level services offered by community health volunteers to national referral hospitals. Facility-based antenatal services are available from Level 2 to Level 6. Therefore, pregnant women can choose to receive care from any facility, although geographic access, cost and personal preferences play a role. Level 2–Level 3 facilities offer basic ANC services such as pregnancy monitoring, blood pressure and urine monitoring, immunisations for pregnant women and human immunodeficiency virus (HIV) testing. High-risk pregnancies are referred to higher levels of care that have resources for laboratory and inpatient management. At all levels, midwives take the lead in offering antenatal services. The current study focused on the population of postnatal mothers from primary care facilities attending one of the two national referral hospitals (Level 6), anonymised here as Referral Hospital B (RH-B). This hospital has over 200 lower-level facilities in its catchment area. Referral Hospital B is a teaching referral hospital. The hospital provides services for up to 10 000 births annually.^23^

### Study population

The target population was all postnatal mothers in RH-B within 42 days of delivery. Only mothers within the hospital at the time of the study were included. Mothers who were too sick to participate at the time of data collection were excluded from the study.

### Sample size

Sample size was based on the methodology for ordinal outcomes in clinical research.^24^ Continuity and coordination were measured on a five-point ordinal scale from ‘strongly disagree’, ‘disagree’, ‘agree’ to ‘strongly agree’. We hypothesised that the odds of near-miss mothers being in the disagree categories would be twice that of women without near miss. Using an allocation ratio of 1:2, we determined that a sample of 89 participants in the near-miss group and 178 in those without near miss would achieve 80% power to detect an odds ratio of 2 when the significance level (alpha) was 0.05 using a two-sided Mann–Whitney *U* test. Although near miss is a relatively rare phenomenon, we considered the computed sample size achievable because near-miss survivors from the catchment population are referred to RH-B for postnatal care, thus increasing the available pool of participants.

### Sampling strategy

Postnatal women were consecutively sampled from the maternal and child health (MCH) clinic during May 2021. To increase the pool of available near-miss survivors, research assistants also visited postnatal inpatient wards where mothers who experienced a severe morbidity were receiving treatment. Mothers were then categorised into those with and without near misses during pregnancy and birth. For obstetric near-miss cases, the inclusion criteria were based on the World Health Organization categorisation.^25^ For the purpose of this study, we used disease-specific and management-specific criteria. Disease-specific criteria included: (1) eclampsia, (2) severe pre-eclampsia, (3) severe postpartum haemorrhage (blood loss of > 1000 mL), (4) severe sepsis and (5) ruptured uterus. Management-specific criteria included women who: (1) received blood transfusion (2) underwent an emergency caesarean section and/or (3) underwent a hysterectomy because of massive haemorrhage.

### Data collection tools and measurement of variables

The data collection instrument consisted of three sections. The first section was on sociodemographic and antenatal visitation characteristics. These included age, marital status, educational level, occupation and distance from usual antenatal clinic. Furthermore, the presence of any non-life-threatening morbidity in pregnancy was assessed to include previous caesarean section, infections, pregnancy-induced hypertension, diabetes mellitus, deep venous thrombosis, premature rupture of membranes, malaria and HIV.

The second section was designed to assess longitudinal continuity by asking women about the number and sequence of antenatal visits, the type of providers seen and the name of the facility for each visit. Using this information, two indices that measure density and dispersion of antenatal visits^26^ were calculated as follows: (1) The *continuity of care (COC) index* measured the dispersion of visits by assigning a higher value to women who visit the same antenatal clinic. For example, a participant scored zero if all four antenatal visits were to a different facility. (2) The *modified continuity index (MCI)* was adjusted for utilisation by assigning a higher value to those with more frequent visits to the same providers. For all indices, a value of 1.0 was considered perfect longitudinal continuity, 0.75–0.99 was high, 0.50–0.74 was medium and below 0.50 was poor.^11^

The third part of the tool measured self-reported continuity and coordination of care using a five-point Likert scale adapted from the Nijmegen Continuity Questionnaire.^27^ This tool was developed in the context of both generalist and specialist medical practice in the Netherlands, among patients with chronic disease. It has since been used in more than 20 studies in chronic care settings, especially in Europe. One strength of this tool is that as items are generic, it can be adapted to various care settings.^27^ We modified the wording to reflect antenatal consultations and added items on sequential coordination of care.

#### Psychometric properties

Content validation of the tool was performed by a group of community health educators in a local Kenyan University (KU). Each expert was asked to score the tool based on five criteria, namely: (1) measurement aim (discriminative vs. evaluative), (2) the target population, (3) the concept being studied and whether the subscale measured the concept of interest, (4) how items were selected and (5) clarity, brevity and interpretability. Cronbach’s alpha was used to assess internal consistency, whilst the intraclass correlation coefficient was used to assess reproducibility. Based on validation and pilot testing, items assessing social support during pregnancy, care navigation and community-based informal caregiving were removed. Cronbach’s alpha for internal consistency was then 0.775, and intraclass correlation coefficient for reproducibility was 0.776. A Cronbach’s alpha of 0.70–0.95 is considered good internal consistency. The final tool consisted of 16 items (Table 3), which we considered a unidimensional tool measuring ‘continuity and coordination of antenatal care’.

### Data collection process

Data were collected over a period of three weeks in May 2021. Five research assistants and the researcher (S.M.M) collected the data. The research assistants were registered nurse-midwives with undergraduate-level knowledge of midwifery and reproductive health. The researcher had a training session with the research assistants to ensure reliable data collection and that ethical considerations were followed. The research assistants obtained informed consent from the mothers and provided the questionnaires if they agreed to participate. The completion of the questionnaires took between 20 min and 30 min. The research assistants were available for clarifications and questions as the mothers completed the questionnaires. The tool was available in English and Kiswahili, and the mothers were free to choose the language that they were most comfortable in.

### Data analysis

The Statistical Package for Social Sciences (SPSS) version 26 was used for data analysis. Analysis was based on complete case analysis. Data entry and checking were conducted by the first author (S.M.). Data analysis was based on the complete case analysis. Distributional assumptions of scores were tested using the Kolmogorov–Smirnoff test and visualisation of QQ plots. Continuity of care and modified continuity of care (MCOC) indices were compared using independent samples Mann–Whitney *U* tests. The Likert scale was analysed as a unidimensional scale. Firstly, each item was summarised using means and standard deviations. A composite score was then computed for near-miss and normal delivery cases. The Mann–Whitney *U* test was then used to test the hypothesis of equality of means for the two groups. To test the effect of longitudinal continuity indices on occurrence of a near miss, continuity of care index (COCI) and MCOCI were entered into a binary logistic regression model adjusted for socio-demographic and antenatal characteristics.

### Ethical considerations

Ethical clearance to conduct this study was obtained from the Stellenbosch University Health Research Ethics Committee (ref. no. S20/02/039 [PhD]), the Moi Teaching and Referral Hospital Ethics Review Committee (ref. no. FAN 0003691) and the National Commission for Science, Technology and Innovation in Kenya (ref. no. NACOSTI/P/21/8398). Ethical consent from the various review committees was received in June and July 2021. All procedures performed in studies involving human participants were in accordance with the ethical standards of the institutional and/or national research committee and with the 1964 Helsinki Declaration and its later amendments or comparable ethical standards. Written informed consent was obtained from all individual participants involved in the study.

## Results

### Descriptive data

Out of the anticipated sample size of 267, 216 (81%) women participated in the study. Of these, five questionnaires were discarded during data cleaning because of incomplete entries. The remaining 211 (79%) consisted of 99 near-miss survivors and 112 women without near miss. The two groups did not differ with regard to age, parity, marital status, education, employment status or distance from the ANC clinic. Near-miss survivors were more likely to have a non-life-threatening morbidity in pregnancy compared with those who did not experience a life-threatening condition (Table 1).

**TABLE 1:** Sociodemographic and antenatal visitation characteristics.

Variable	Near-miss cases *N* = 99		Without near miss *N* = 112		*p*
	*n*	%	*n*	%	
**Marital status**					0.325
Single	29	43.9	37	56.1	
Married	70	48.3	75	51.7	
**Education**					0.432
Secondary or tertiary	78	45.6	93	54.4	
Primary or no education	21	52.5	19	47.5	
**Employment**					
Unemployed	43	45.3	52	54.7	0.662
Formal or self-employed	56	48.3	60	51.7	
**Distance from ANC clinic**					
Above 10 km	43	50.0	43	50.0	0.395
Below 10 km	51	44.0	65	56.0	
**Non-life-threatening morbidity** †					
At least one morbidity	81	62.8	48	37.2	< 0.001
No morbidity	18	22.0	64	78.0	

ANC, antenatal care.

† Includes previous caesarean section, infections, pregnancy-induced hypertension, diabetes mellitus, deep venous thrombosis, premature rupture of membranes, malaria, HIV.

### Longitudinal continuity

Overall mean COC index in the study population was 0.76 (s.d. = 0.36). One hundred and twenty-eight (66.30%) participants had a perfect longitudinal continuity index (COCI = 1.00), one participant had high (COC 0.75–0.99), three participants had medium (COC 0.55–0.74) and 61 had poor continuity (COC < 0.55). Near-miss survivors had statistically significant lower continuity indices as compared to those without near miss (Table 2).

**TABLE 2:** Comparison of longitudinal continuity indices between normal delivery and near-miss cases.

Measure of longitudinal continuity	Without near miss *N* = 102		Near-miss cases *N* = 91		MWU	*p*
	Median	95% CI	Median	95% CI		
Continuity of care index	0.80	0.72–0.91	0.70	0.55–0.75	3920.5	0.027
Modified continuity of care index	0.73	0.65–0.84	0.62	0.55–0.72	3022.0	0.034

MWU; Mann–Whitney *U* test; CI, confidence interval.

There was no statistically significant difference between the groups in the number of ANC visits (81.0% in the near-miss group versus 89.6% among those without near miss). As a visit to a different facility may be because of a referral, we explored whether women switched to a higher, similar or lower-level facility in the health system. The assumption being that switching to the same level or lower-level facility may be because of reasons other than referral. Five (5.5%) near-miss survivors switched to the same or lower-level facilities compared with none (0.0%) among uncomplicated cases. Sixteen women (55.2%) switched to higher-level facilities among those without near miss compared with 13 (44.8%) in the near-miss group (*p* = 0.127).

### Self-reported relational continuity and sequential coordination of care

There was no significant difference in the mean composite score for continuity and sequential coordination between the two groups (Table 3). On individual items, there was no significant difference between the two groups on 12 of 16 items. Mothers with normal delivery were significantly more likely to report that their care was well coordinated and that the provider at the higher level of care sent them back to their usual provider. Mothers with near misses were significantly more likely to report that their provider knew their previous medical and obstetric history and that their visit to the higher level was organised by the local clinic.

**TABLE 3:** Comparison of self-reported continuity and coordination of care between uncomplicated cases versus near-miss survivors.

Item	Subdomain	Without near miss			Near-miss cases			MWU	*p*
		*n*	Mean	s.d.	*n*	Mean	s.d.		
I was seen by the same caregiver, doctor or midwife throughout pregnancy	Longitudinal continuity	112	2.90	1.4	97	2.80	1.60	5255.50	0.677
I had confidence in the professional ability of my antenatal care provider	Relational continuity	110	4.10	0.8	99	4.20	0.60	5736.50	0.461
I believed that my provider cared for me	Relational continuity	111	4.30	0.7	97	4.20	0.70	5240.00	0.710
I felt comfortable consulting my health provider about my doubts	Relational continuity	110	4.10	0.7	99	4.20	0.70	5637.50	0.630
My health provider understood what I told him or her	Relational continuity	112	4.10	0.6	98	3.90	0.70	5004.50	0.221
My ANC provider was flexible and adaptable to my changing needs	Relational continuity	110	4.00	0.7	99	4.10	0.90	5499.00	0.947
My provider understood my cultural, and family needs	Relational continuity	110	4.10	0.7	97	4.10	0.90	4715.00	0.124
I believed that the professionals attending to me knew my previous medical and obstetric history	Informational continuity	109	3.80	0.9	99	4.20	0.90	6013.00	0.046
The information my provider gave me was easy to understand	Informational continuity	112	4.10	0.9	98	4.10	0.80	5482.50	0.898
I had a positive communication experience with my providers	Relational continuity	96	4.10	0.8	96	4.10	0.80	5130.00	0.497
When referral was needed, the referral process was clear and easy to follow	Sequential coordination	79	4.00	0.9	83	4.00	1.00	3456.00	0.526
In case of admission, I was given a clear plan to follow after discharge	Sequential coordination	90	3.90	1.0	94	3.70	1.00	3942.00	0.396
The provider from the higher facility always sent me back to my usual provider	Sequential coordination	89	4.00	1.1	93	3.60	1.10	3574.00	0.006
I believe that the care I received was well coordinated	Sequential coordination	99	4.30	0.7	97	3.90	0.70	4103.00	0.050
My visit to a higher-level facility was arranged at my local antenatal clinic	Sequential coordination	90	3.40	1.2	91	3.80	1.20	4831.50	0.030

**Total score on scale items**		**72**	**4.40**	**0.4**	**68**	**4.00**	**0.46**	**2154.00**	**0.226**

MWU, Mann–Whitney *U* test; ANC, antenatal care; s.d., standard deviation.

### Multivariable analysis

In crude regression analysis, every unit increase in COCI scores was associated with a decrease in the odds of a near miss (OR = 0.430, *p* = 0.038) seen in Table 4. Conversely, multiparity (OR = 8.87, *p* = 0.004), presence of a non-life-threatening morbidity (OR = 6.00, *p* = 0.001) and higher number of ANC visits (OR = 1.25, *p* = 0.040) were associated with increased odds of a near miss. In adjusted analysis, the effect of COCI was not apparent (aOR = 0.81, *p* = 0.732). Women who had at least one non-life-threatening morbidity during pregnancy were four times more likely to experience a near miss (aOR = 4.343, *p* = 0.001) after adjusting for all other variables.

**TABLE 4:** Factors associated with obstetric near miss.

Variable	Reference	Crude OR	95% CI		*p*	aOR	95% CI		*p*
			Lower	Upper			Lower	Upper	
Multipara	Primiparous	8.87	1.99	39.52	0.004†	5.27	0.52	52.67	0.157
Primary or no education	Secondary or tertiary	1.31	0.66	2.62	0.430	1.11	0.36	3.47	0.870
Single	Married	1.19	0.66	2.13	0.540	1.01	0.36	2.86	0.980
Non-life-threatening morbidity in pregnancy†	None	6.00	3.18	11.30	0.001†	4.34	1.77	10.68	0.001†
Age	Continuous	1.01	0.97	1.10	0.610	1.02	0.94	1.10	0.625
Number of ANC visits	Discrete	1.25	1.00	1.55	0.040†	2.03	0.73	5.65	0.178
COC index	Continuous	0.43	0.19	0.95	0.038†	0.81	0.26	2.60	0.732

COC, continuity of care; ANC, antenatal care; aOR, adjusted odds ratio; OR, odds ratio; CI, confidence interval.

† Includes previous caesarean section, infections, pregnancy-induced hypertension, diabetes mellitus, deep venous thrombosis, premature rupture of membranes, malaria, HIV.

## Discussion

### Key findings

Obstetric near-miss cases had lower longitudinal continuity indices in bivariate analyses, but the association was not apparent after adjusting for all variables in the study. The presence of at least one non-life-threatening morbidity in pregnancy was the strongest predictor of a near miss. Mothers with normal delivery were significantly more likely to report that their care was well coordinated and that the provider at the higher level of care sent them back to their usual provider. Mothers with near misses were significantly more likely to report that their provider knew their previous medical and obstetric history and that their visit to the higher level was organised by the local clinic.

### Discussion of key findings

Our findings on COC indices are similar to a recent study conducted in Ghana that found indices of between 0.70–0.85.^11^ They, however, differ from other studies in the Netherlands and Belgium, which show relatively low longitudinal continuity indices of between 0.40 and 0.50.^12,13,14^ In these countries, there is significant heterogeneity in antenatal visitation because of the model of service delivery. For example, in the Netherlands, general practitioners usually transfer care of pregnant women to obstetricians and midwives during the third trimester, thus explaining the low COCI as compared to Ghana and Kenya.^13^

The association of longitudinal continuity and near miss in bivariate but not adjusted analysis differs from a study in Ghana, which showed an association between low continuity indices and caesarean delivery in adjusted analysis.^11^ Notably, the aforementioned study used a much larger sample size (*n* = 14 350) derived from insurance data. It is possible that our much smaller size constrained our ability to find associations. Longitudinal continuity fosters relationships with health providers such that they can recognise and refer problems earlier.^11,28^ However, it may not reflect other factors in the causal pathway to near miss such as competency and confidence of health providers, infrastructure, referral mechanisms and poor adherence of women to advice, among other factors.^29^ In health systems that promote many antenatal contacts, such as Kenya, there may be a need to move beyond focusing on continuity to addressing the extent to which these visits influence the quality of care. On the other hand, low continuity may not necessarily confer negative consequences, as women who change facilities may do so in search of a better quality of care.^18^ Therefore, policy changes with regard to the number of antenatal visits should be accompanied by improvement in the quality of facility services. For example, the WHO publication on *recommendations on ANC for quality pregnancy experience* has proposed that countries revert to the eight-visit schedule from the four-visit schedule under the FANC model.^6^ Concurrent efforts at strengthening coordination mechanisms and accountability at the facility level may be more fruitful in the long term.

Near-miss survivors felt that their care was not well coordinated between levels. In a previous scoping review, mothers reported that a smooth transition from one level to another with timely information is one of their priorities for a positive pregnancy experience.^30^ Client’s notions of uncoordinated care may arise from delays in referral systems, long waiting times, lack of specialised services and complicated billing systems, among others. Quality of communication between staff may also reinforce the impression of poorly coordinated care as well. In a previous study in the Netherlands, mothers attending ANC clinics were able to pick up tensions and lack of communication among facility staff, reinforcing the impression of poorly coordinated care.^19^ Coordination of care is an important supply-side factor in maternal health. Innovative system interventions for improving care coordination need scaling up. This includes the use of care navigators and adherence to indicators for coordination.^31,32,33^

In the present study, the presence of at least one non-life-threatening morbidity in pregnancy was predictive of a near miss. Early identification and management of morbidities during ANC contact are influenced by various factors such as adherence to clinical guidelines among health providers, as well as patient factors that may hinder adherence to practices that promote early intervention.^34^

### Strengths and limitations

Weaknesses associated with cross-sectional studies include limited external validity, lack of temporality and inability to infer causality. The study was carried out in a large tertiary hospital, which may not generalise to mothers in lower-level facilities. The study used self-reported measures of continuity and coordination that may differ from more objective ways of measuring continuity and coordination. Our inclusion criteria included up to 1-month postpartum. Therefore we may have missed out mothers who developed complication up to 42 days postpartum based on the WHO definition of near miss. As we only analysed questionnaires with complete responses, it is possible that if those with missing responses were not random, potential bias may have been introduced. Our response rate was 79%, which was lower than anticipated. It is possible that a higher response rate may have yielded different results. We used consecutive sampling that may lead to biases associated with non-probability sampling methods. We also recognise the potential for recall bias because near-miss survivors are more likely to pick up and recall events during ANC. Furthermore, mothers’ responses may be limited by different interpretations of questionnaire items. We did not assess reasons for changing facilities, which may have been more informative given our finding on lower continuity scores among near-miss cases. Finally, we acknowledge that statistically significant results on continuity and coordination scores may not necessarily translate to practical or clinical importance.

Despite these limitations, we consider this study an important addition to the literature in this area for several reasons. Firstly, we incorporate objective measures of longitudinal continuity, which is not common. We also operationalised continuity into longitudinal, relational and informational continuity, which is more practical given that each of them may have different antecedents.

### Implications for research and practice

It would be informative to investigate whether mothers who switch facilities in search of higher quality care (and therefore have lower longitudinal continuity indices) actually end up receiving better care. If relational factors play a role, it would be important to investigate the level of longitudinal continuity that is sufficient for the development of meaningful provider–mother relationships. This also means paying more attention to intermediate outcomes by developing more standardised tools for measuring maternal preferences with care.^5^ These may include a preference for a given type of provider, place of antenatal consultation and preferred mode of delivery, among others. The recent Cochrane review, which showed better perinatal outcomes in midwife-led continuity models is of interest and may need to be replicated in low-income settings because the original study was based on studies from high-income settings.^5^

More in-depth (preferably qualitative) studies are needed among expectant mothers sub-Saharan Africa about their perception of ‘coordination of care’ and what they feel is important for them. Providers need to focus more on aspects of care coordination that are readily discernible by the clients. This includes interprofessional communication, feedback loops and willingness to collaborate. Participatory approaches for improving coordination using co-design methods (meetings, joint planning, reflection, feedback) have been piloted in Latin America^35^ and may be adopted in Kenya and similar settings.

Non-life-threatening morbidities in pregnancy remain important predictors of the eventual occurrence of a near miss and mortality; therefore, the factors that hinder their timely recognition and referral require more attention and research. Adoption of early warning systems, such as the Maternal Early Warning system (MEOWs), may improve recognition of potential progression to severe outcomes in the third trimester.^36^

## Conclusion

The results of this study show that while longitudinal and relational COC is important during the antenatal period, the presence of a non-life-threatening condition in pregnancy remains the most important predictor of the occurrence of a near miss. It also appears that expectant mothers perceive that their care is not well coordinated across levels. This implies a need for providers to strengthen coordination, maybe through more participatory techniques. Future studies may focus on determining the optimum level of longitudinal continuity necessary for fostering mother–provider relationships as intermediate factors. At the same time, systems for early identification and management of non-life-threatening morbidities in pregnancy are crucial.
